# Present Scenario and Future Landscape of Payloads for ADCs: Focus on DNA-Interacting Agents

**DOI:** 10.3390/ph17101338

**Published:** 2024-10-07

**Authors:** Barbara Valsasina, Paolo Orsini, Chiara Terenghi, Alberto Ocana

**Affiliations:** 1 Nerviano Medical Sciences, Viale Pasteur 10, 20014 Nerviano, Italy; 2 Department of Pharmaceutical Sciences, University of Milan, Via Mangiagalli 25, 20133 Milan, Italy; 3 Experimental Therapeutics Unit, Hospital Clínico San Carlos (HCSC), Instituto de Investigación Sanitaria San Carlos (IdISSC), 2546 Madrid, Spain; 4 START Madrid-FJD, Hospital Fundación Jiménez Díaz, 2546 Madrid, Spain; 5 Breast Cancer, Centro de Investigación Biomédica en Red en Oncología (CIBERONC), 2546 Madrid, Spain

**Keywords:** ADC, payload, DNA damage, cancer, anthracycline, duocarmycin, PBD, calicheamicin

## Abstract

ADCs have emerged as a promising class of therapeutics, combining the targeting specificity of monoclonal antibodies with the cytotoxic potency of small-molecule drugs. Although the majority of approved ADCs are still based on microtubule binder payloads, the recent success of topoisomerase I inhibitors has revitalized interest in the identification of novel agents overcoming present limitations in the field including narrow therapeutic window and chemoresistance. The success of DNA binders as payload for ADCs has been very limited, up to now, due, among other factors, to high hydrophobicity and planar chemical structures resulting in most cases in ADCs with a strong tendency to aggregate, poor plasma stability, and limited therapeutic index. Some of these molecules, however, continue to be of interest due to their favorable properties in terms of cytotoxic potency even in chemoresistant settings, bystander and immunogenic cell death effects, and known combinability with approved drugs. We critically evaluated several clinically tested ADCs containing DNA binders, focusing on payload physicochemical properties, cytotoxic potency, and obtained clinical results. Our analysis suggests that further exploration of certain chemical classes, specifically anthracyclines and duocarmycins, based on the optimization of physicochemical parameters, reduction of cytotoxic potency, and careful design of targeting molecules is warranted. This approach will possibly result in a novel generation of payloads overcoming the limitations of clinically validated ADCs.

## 1. Introduction

Antibody Drug Conjugates (ADCs) are antibody-based macromolecular complexes consisting of three primary components: antibodies targeted to cell membrane proteins highly expressed in tumor cells, linkers designed to remain stable in the bloodstream while releasing the cytotoxic payload within tumor cells, and the payloads themselves, which are cytotoxic agents causing tumor cell death. ADCs have demonstrated potent antitumor activities against treatment-refractory cancers in clinical settings leading to the US Food and Drug Administration (FDA) agency granting regulatory approval for 12 ADCs, 6 for use in hematological malignancies and 6 for use in solid tumors, as of September 2024 (Table 1). It is worth noting that 8 of these 12 ADCs have been approved in the last 5 years, indicating a maturation of the field over time further evidenced by the presence of an additional 256 ADCs currently undergoing clinical trials [1].

Given that ADCs comprise distinct elements, harmonizing these components is essential to achieve optimal results in terms of efficacy, safety profile, and pharmacokinetic properties.

**Figure 1:**
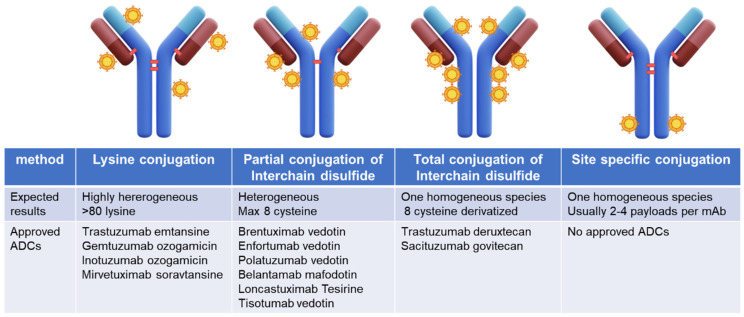
Conjugation methods for ADC development and approved ADC development with the reported method.

First-generation ADCs such as gemtuzumab ozogamicin [2], approved by the FDA in 2000 for AML patients, voluntarily withdrawn from the market by Pfizer due to safety issues in 2010, and re-approved by the FDA in 2017, trastuzumab emtansine, approved by the FDA in 2013 for HER2-positive advanced or metastatic breast tumors [3], and inotuzumab ozogamicin [4], approved in 2017 for ALL and B-cell lymphomas were developed with a lysine conjugation approach resulting in highly heterogeneous products [5]. Recently, the introduction of interchain disulfide conjugation has reduced heterogeneity in the second-generation ADCs. In the case of trastuzumab deruxtecan [6] and sacituzumab govitecan [7], which bear topoisomerase inhibitor payloads, highly homogeneous third-generation ADCs have been achieved through complete cysteine interchain derivatization reaching a Drug Antibody Ratio (DAR) of 8 without negatively impacting final ADC properties (Figure 1).

More recently emerged site-specific conjugation technologies couple payloads to specifically defined sites in antibody molecules [8] including cysteine, glutamine, unnatural amino acids, short peptide tags, and glycans, generating highly homogeneous ADCs with the desired DAR. However, no ADCs generated by site-specific conjugation have reached FDA approval so far.

Furthermore, the initial ADCs were designed with uncleavable linkers [5] that require complete antibody digestion to release the payload still bound to an antibody fragment. In contrast, new-generation ADCs rely on protease cleavable linkers [9]. Protease cleavage releases the free payload from the antibody, allowing it to diffuse to surrounding tumor cells promoting the bystander effect, thereby causing target-negative cell killing. This is a fundamental mechanism to be considered in the case of solid tumors where heterogeneous expression of antigen is observed [10].

The optimization of all required parameters and introduction of a different payload allowed, indeed, the homogeneous DAR 8 trastuzumab deruxtecan, with a cleavable linker conjugated to mAb interchain cysteines, to reach regulatory approval even in indications where trastuzumab emtansine, based on the same antibody but containing a microtubule-binding agent payload with lysine conjugation and uncleavable linker, failed to obtain positive results. In the Destiny- Breast 4 phase III clinical study of trastuzumab deruxtecan in metastatic breast cancer HER2-low, HR-positive, or triple-negative progression-free survival was indeed doubled compared to the physician’s choice arm, irrespective of patient characteristics [11].

Resistance to topoisomerase-I-inhibitor and tubulin-binder-based ADCs is, however, emerging. Additionally, a higher therapeutic window is expected for next-generation ADCs as ADCs based on tubulin binding agents cause peripheral neuropathy and hematolymphopoietic-system-related side effects, limiting their combinability, while topoisomerase inhibitors exhibit strong gastrointestinal side effects resulting in dose reduction or treatment interruption. Up to 14% of patients treated with trastuzumab deruxtecan also experienced interstitial lung disease with several fatal cases reported [12].

Strategies to overcome resistance and improve the safety profile thereby allowing for greater combinability are urgently needed. These strategies can be addressed by considering the different mechanisms of resistance and the safety profile of alternative payloads.

## 2. Mechanism of Resistance

**Figure 2:**
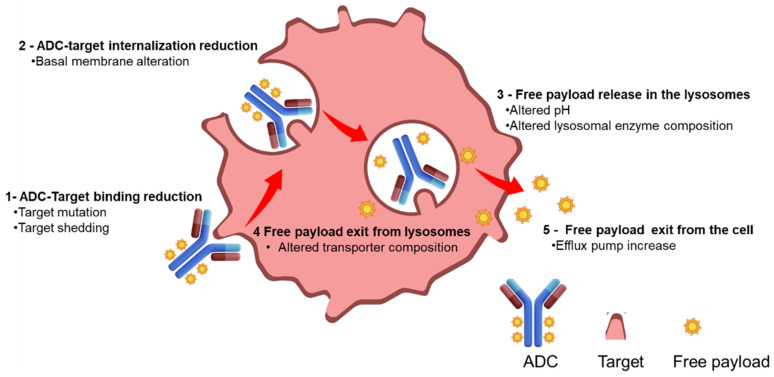
Reported mechanism of resistance for ADCs.

Acquired or primary resistance to ADC therapy has to be considered a multifactorial event due to the different steps involved in the ADC mechanism of action [13]: antigen recognition, internalization, payload release inside the lysosome, and trafficking inside the cell to reach the target (Figure 2).

The resistance mechanism can be associated with an altered target expression, a modified ADC trafficking inside the cells, or payload-related properties. A multifactorial resistance mechanism is corroborated by a recent bioinformatic work of Bosi et al. aimed to assess the expression of ADC targets and potential downstream determining factors of activity across pan-cancer and normal tissues. The analysis identified 59 genes potentially implicated in ADC response; 14 of these were associated with resistance and 45 with sensitivity involving internalization, linker lysis, endosomal trafficking, and payload metabolism [14].

A reduction in target expression on the cell membrane as well as mutation in the extracellular domain of the target protein or antigen shedding can be observed in refractory cancers resistant to targeted monoclonal antibody therapies [15]. A similar mechanism of resistance has been observed for some antibody–drug conjugates [16]. As an example, preclinical studies of acquired resistance to trastuzumab–maytansinoid ADC showed that primary mediators of resistance upon chronic tumor cell treatment with T-DM1 were increased ABCC1 protein and reduced HER2 antigen expression [17]. The re-expression of HER2 antigen in the same cell line reverted the resistance, confirming a reduced-target-expression-mediated mechanism. The use of a different payload can overcome target-alteration-driven resistance as demonstrated by clinical studies with trastuzumab deruxtecan or trastuzumab duocarmazine. These studies showed responses in T-DM1-pretreated patients due to higher payload potency, bystander effect, and cleavable linkers able to act in heterogeneous and lower-target-expressing tumors [18].

An increase in the cellular basement membrane has been proposed as a resistance mechanism to ADC internalization. Abnormal endosomal transit and the absence or reduced proteolytical activity in the lysosomes, due to a change of pH, were also evidenced in a recent preclinical work about T-DM1 resistance. ADC trafficking can be facilitated by the use of alternative linkers relying on different release mechanisms such as redox or acidic release vs. release mechanisms purely based on lysosomal protease activity.

Resistance mechanisms involving specific payloads have been widely reported. Trastuzumab deruxtecan efficacy in mouse tumor models resistant to T-DM1 was clearly observed, while, conversely, in NCI-N87, a HER2-positive gastric xenograft model with induced resistance to trastuzumab deruxtecan and tumor regression upon treatment with an MMAE-based trastuzumab ADC was observed.

Documented resistance to topoisomerase I inhibitor ADCs indicated expression of topoisomerase I [14] itself or changes in the downstream signaling mechanisms as the main causes. In the Daisy II clinical study [19], resistance to trastuzumab deruxtecan has been related to a specific gene mutation named SLX4. SLX4, a protein involved in DNA damage response [20], was found mutated in 20% of breast cancer patients resistant to trastuzumab deruxtecan. Moreover, SLFN11 alterations were recently implicated in resistance to topoisomerase-I-inhibitor-based ADCs. SLFN11 is a putative DNA/RNA helicase involved in irreversible replication block and cell death and previously correlated to sensitivity to camptothecin derivatives [21]. Phase I clinical studies combining trastuzumab deruxtecan with valemetostat, an EZH2 inhibitor possibly reverting EZH2-mediated epigenetic downregulation of SLFN11, are ongoing in Japan [22].

The increased efflux of the ADC payload, mediated by ATP-binding-cassette transporters, is one of the most common mechanisms of resistance. The transporters were noted to have increased expression, up to 20–50 times higher, in cells resistant to T-DM1 as opposed to the parent cells. This mechanism was observed in models of AML cells as a means of resistance to gemtuzumab ozogamicin (GO) [23]. Similarly, patients with lower levels of ABCB1, which encodes an ATP-binding cassette, had an improved response to GO. A preclinical breast cancer mouse model, treated with an anti-nectin-4-directed ADC known as N41mab–vcMMAE, was analyzed using RNAseq after 9 months of treatment with the ADC. In this model, there was upregulation in ABCB1. When the ADC was administered in combination with the P-gp pharmacologic inhibitor tariquidar, a rapid treatment response was seen, which was substantially better tolerated than the combination of tariquidar and docetaxel [13].

As previously highlighted, the use of a different payload could successfully revert observed chemoresistance, and the availability of payloads with different mechanisms of action, sensitivity to the MDR system, and physicochemical properties will allow better exploitation of ADC technology.

Payloads with different mechanisms and structures have been evaluated across the years, including cytotoxins, immunostimulating agents, PROTAC, oligonucleotides, and targeting agents [24]. The core of this review focuses on DNA-interacting payloads.

## 3. Current Status of ADCs Containing DNA-Binding Payloads

Different classes of DNA-interacting ADCs containing minor groove binders and alkylating agents, DNA crosslinkers, and topoisomerase II inhibitors have reached clinical trials, with duocarmycin, calicheamicins, PBD dimers, and anthracyclines being the main represented chemical classes. Indolino benzodiazepines (IGNs), a chemical class derived from PBD, have been considered as well, being initially proposed as a less toxic version of PBD with DNA alkylation properties.

**Figure 3:**
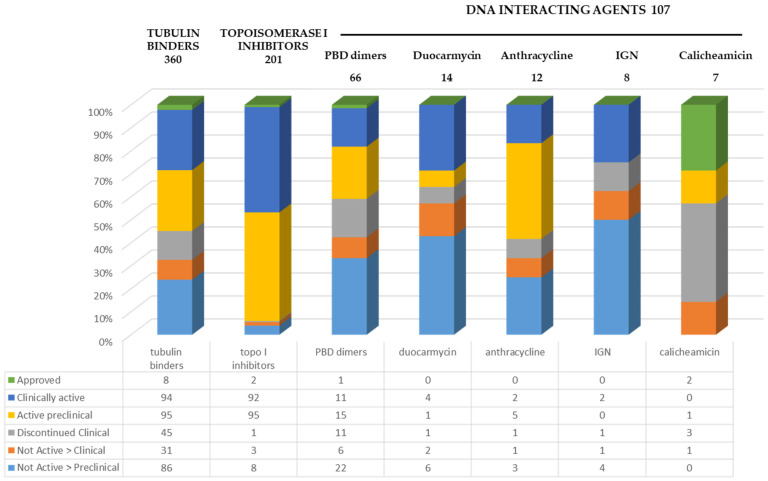
ADC advancement status based on payload mechanism of action [1].

Only 3 out of the 12 currently approved ADCs contain DNA-interacting molecules (Table 1) and all of these are approved in the treatment of hematological malignancies, namely the previously mentioned calicheamicin-based gemtuzumab ozogamicin and inotuzumab ozogamicin and the more recent PBD-dimer-based loncastuzumab tesirine [25]. A total of 107 ADCs containing DNA-binding molecules are reported to be at different development stages, compared to 360 tubulin binders and 201 topoisomerase I inhibitors, as of September 2024 (Figure 3) [1].

Among the different classes of DNA-interacting payloads, PBD, with a DNA crosslinking mechanism of action, is the most explored with 66 ADCs developed but only 1, loncastuzumab tesirine, approved, 11 ADCs clinically active, and 15 under preclinical development. The Indoline benzodiazepine (IGN) class of payloads has up to now proved to be the less successful class with 13% of the products abandoned at the clinical stage and 62% at the preclinical stage, no approved ADC, and only 2 clinically active. Anthracyclines, at present, represent the chemical class with more compounds still undergoing development either at a clinical or preclinical stage (58%). Similar statements can be made about the duocarmycin class with 36% of currently active ADCs at either a clinical or preclinical stage.

The total number of ADCs considered in the calculation is, however, low, namely, 8 ADCs for IGN, 12 for anthracyclines, and 14 for duocarmycins.

The poor exploration of these chemical classes compared to tubulin binders and topoisomerase I inhibitors could be related to a difficult synthetic process or to the generated molecules not being suitable for conjugation with mAb, namely compounds poorly soluble in aqueous solutions or with an extremely rigid structure causing distortion of mAb upon conjugation and protein unfolding.

## 4. DNA-Binding Payload Background and Clinical Results

ADCs containing DNA binding molecules have reached clinical trials during the years being the ADC containing Calicheamicin the first to reach regulatory approval followed more recently by the approval of pyrrolobenzodiazepine dimer-based ADC Loncastuximab Tesirine. Other chemical classes of DNA binders have been tested as payload for ADCs during the years but up to now no additional ADC has reached approval (Table 2).

### 4.1. Calicheamicin

Calicheamicin [26] is a highly potent antitumor antibiotic that targets the minor groove of DNA causing double-strand breaks. Seven calicheamicin-based ADCs have been explored either clinically or preclinically with two receiving FDA approval, namely, the CD33 ADC gemtuzumab ozogamicin and the CD22 ADC inotuzumab ozogamicin, approved for acute myeloid leukemia (AML) and acute lymphoblastic leukemia (ALL), respectively.

Gemtuzumab ozogamicin and inotuzumab ozogamicin consist of humanized IgG4 antibodies, targeting CD33 and CD22, respectively. These antibodies are conjugated to calicheamicin via an acid-labile linker through lysine conjugation, resulting in a heterogeneous mixture with an average drug-to-antibody ratio (DAR) of 2–3 but containing over 50% free antibody.

Both drugs demonstrated favorable preclinical profiles; however, sensitivity to efflux pumps and consequently reduced activity in p-glycoprotein overexpressing cells was noted [23].

Gemtuzumab ozogamicin was initially approved in 2000, but it was voluntarily withdrawn from the market in 2010 due to increased mortality and high incidence of hepatic veno-occlusive disease (VOD). It was re-approved for AML following new clinical trial data that demonstrated efficacy with an acceptable safety profile using a dose fractionation schedule and treating only patients with newly diagnosed AML [27].

Inotuzumab ozogamicin [4] was approved by the FDA in August 2017 for adult patients with relapsed/refractory B-cell precursor acute lymphocytic leukemia (ALL). This approval was based on data from a randomized (1:1), open-label, international, multicenter phase III study in 326 patients with Philadelphia chromosome-negative or Philadelphia chromosome-positive relapsed or refractory B-cell precursor ALL. Following the gemtuzumab ozogamicin experience, dose fractionation was also implemented recommending an initial cycle dose of 1.8 mg/m², administered as three divided doses on day 1 (0.8 mg/m²), day 8 (0.5 mg/m²), and day 15 (0.5 mg/m²). The median overall survival (OS) was 7.7 months with inotuzumab ozogamicin and 6.2 months with SoC, with 2-year OS rates of 22.8% and 10.0%, respectively. The most frequent all-grade and grade 3 or higher adverse events in both arms were hematologic. Veno-occlusive disease (VOD)/sinusoidal obstruction syndrome (SOS) was more frequent with inotuzumab ozogamicin then gemtuzumab ozogamicin (23 of 164 [14.0%] vs. 3 of 143 [2.1%]) [28].

All clinically and preclinically evaluated calicheamicin-based ADCs utilize a hydrazone linker designed to release the cytotoxic payload under acidic conditions in lysosomes. Recent studies, however, have suggested that the instability of hydrazone linkers is a potential liability with plasma release of the drug reducing efficacy and increasing the probability of off-target side effects [29].

ABBV-011 [30] is the only calicheamicin-bearing ADC prepared with a different process. ABBV-011 consists of an anti-SEZ6 antibody conjugated to calicheamicin via engineered cysteines with a cleavable linker yielding a homogeneous molecule with a DAR of approximately 2. ABBV-011 progressed to phase I clinical trials with 132 patients enrolled, performing dose escalation every three weeks at doses of 0.6 mg/kg (*n* = 4), 1.2 mg/kg (*n* = 5), 1.6 mg/kg (*n* = 5), and 2.0 mg/kg (*n* = 9). Delayed-onset hepatotoxicity (hyperbilirubinemia, GGT increase) limited long-term dosing at doses above 1.2 mg/kg. Consequently, 1 mg/kg was selected for dose expansion and 44 patients were treated every three weeks.

Dose expansion results [31] indicated that grade 3 treatment-emergent adverse events (TEAEs) occurred in 45% of patients with the most frequent being fatigue, thrombocytopenia, and neutropenia (10% each). One grade 4 TEAE of dyspnea was reported. Hepatotoxicity was observed, including grade 2 or higher TEAEs of hyperbilirubinemia (18%), increased gamma-glutamyltransferase (8%), ascites (5%), VOD (3%), and portal hypertension (3%).

The confirmed objective response rate was 25% (10 partial responses), with a median duration of response of 4.2 months. The clinical benefit rate (CBR) was 65% (10 partial responses and 16 stable disease) and the CBR lasting >12 weeks was 43%. The median progression-free survival was 3.5 months. In August 2023, ABBV-011 was removed from Abbvie’s early pipeline indicating the company’s decision to discontinue its development.

### 4.2. Pyrrolobenzodiazepine Dimers (PBD)

Anthramycin was the inaugural member of the pyrrolobenzodiazepine (PBD) class of antitumor antibiotics, discovered in the 1960s [32]. PBDs function by selectively binding and alkylating the DNA minor groove. Notably, synthetic PBD dimers exhibit heightened cytotoxicity due to their capacity to form two covalent bonds, enabling DNA cross-linking with antiproliferative efficacy at femtomolar concentrations. Given their potent activity, PBD dimers have been explored as payloads for antibody–drug conjugates [33], and generated ADCs achieved complete tumor regressions in multiple preclinical in vivo models and effectively targeted low-copy-number antigens. Furthermore, PBD dimers can target slowly proliferating cells, including cancer stem cells or tumor-initiating cells, which is crucial for sustained tumor regression and prevention of recurrence [34].

P-glycoprotein expression can modulate the activity of some PBD dimers in vitro and in vivo, depending on the specific PBD structure. Many potent PBD dimers are not significant P-glycoprotein substrates, providing a significant advantage over other natural-product-derived ADC warheads. ADCs with cleavable linkers that deliver PBD dimers have demonstrated efficient bystander cell killing both in vitro and in vivo. Over 25 PBD dimer-based ADCs have progressed to clinical trials [35], with a similar number explored preclinically. Two notable PBD dimers, talirine and tesirine, have been developed as payloads.

SGN-CD33A, the first ADC containing talirine [36], is an engineered monoclonal antibody (mAb) with cysteine at position 239 on the heavy chain for site-specific conjugation. SGN-CD33A exhibited superior preclinical efficacy compared to gemtuzumab ozogamicin, achieving complete and durable responses in subcutaneous acute myeloid leukemia (AML) xenograft models with a single dose as low as 100 μg/kg. Encouraging preclinical results led to clinical studies in AML [36], both as a single agent and in combination with azacytidine. A global, randomized, double-blinded, placebo-controlled phase 3 trial named CASCADE was initiated in 2016 but terminated in June 2017 due to a higher mortality rate, including fatal infections, in the SGN-CD33A arm. Seattle Genetics also evaluated other talirine-containing ADCs clinically; however, modest single-agent activity led to the discontinuation of further development.

The development of talirine highlighted the challenges of limited aqueous solubility and potential aggregation during antibody conjugation, prompting the creation of novel PBD-dimer-containing payloads that could be conjugated in aqueous buffers with minimal aggregation [37].

Rovalpituzumab tesirine [38] is an ADC composed of a humanized IgG1 antibody targeting delta-like 3 protein (DLL3), conjugated to tesirine. DLL3, an atypical Notch receptor ligand, may function as an oncogenic driver in high-grade neuroendocrine tumors, including small-cell lung cancer (SCLC). In patient-derived xenograft (PDX) models, rovalpituzumab tesirine showed efficacy correlated with DLL3 expression, effectively targeting DLL3-expressing tumor-initiating cells in SCLC and large-cell neuroendocrine carcinoma. Although phase I studies showed promising single-agent activity with a manageable safety profile, subsequent phase II and III studies (TRINITY and TAHOE) demonstrated limited antitumor activity and higher adverse event rates, leading to early termination [39,40].

Despite setbacks, tesirine-containing ADCs remain of interest, as evidenced by the recent approval of loncastuximab tesirine for hematological malignancies. Loncastuximab tesirine [41] targets human CD19 with a humanized IgG1 antibody, stochastically conjugated to tesirine with a DAR of 2.3. It was evaluated in patients with relapsed/refractory B-cell non-Hodgkin lymphoma (NHL). A phase 2 trial (LOTIS-2) demonstrated an overall response rate (ORR) of 48.3%, with a complete response (CR) rate of 24.1%. The FDA approved loncastuximab tesirine on 23 April 2021, based on these results, marking a significant advancement in the treatment of B-cell malignancies. The trial also reported a median time to response of 1.3 months and a median duration of response of 10.3 months. Common grade equal to or above 3 treatment-emergent adverse events included neutropenia, thrombocytopenia, increased gamma-glutamyltransferase, and anemia [42].

### 4.3. Indolino Benzodiazepine Dimers (IGN)

Indolino benzodiazepines or IGNs, are ultra-potent molecules that alkylate DNA via a single imine moiety within the dimer structure, distinguishing them from PBD dimer drugs [43].

Pivekimab Sunirine (IMGN632) is a CD123-targeting ADC consisting of an anti-CD123 antibody linked, through a peptide linker, to the IGN compound sunirine. Preclinical evaluations have shown that IMGN632 exhibits significant activity against AML, blastic plasmacytoid dendritic cell neoplasm (BPDCN), and ALL models, with a broad therapeutic index in animal models, and a 150-fold differential cytotoxicity in AML patient samples compared to normal hematopoietic progenitors [44,45]. The pronounced sensitivity of BPDCN-patient-derived xenografts to IMGN632 has also been demonstrated [46].

In the dose escalation part of the Phase I/II study, a recommended phase 2 dose (RP2D) of 0.045 mg/kg administered every 3 weeks (Q3W) was established. This study reported a 40% ORR rate in relapsed and refractory de novo AML patients treated at the RP2D with manageable toxicity. Ongoing studies are currently investigating the combination of IMGN632 with venetoclax and azacytidine.

TAK-164 [47], another IGN ADC targeting Guanylyl cyclase C (GCC), which is highly expressed in several gastrointestinal malignancies, underwent a phase I clinical trial to evaluate efficacy and tolerability (NCT03449030). Thirty-one patients with GCC-positive, advanced gastrointestinal cancers received intravenous TAK-164 on day 1 of 21-day cycles in a dose escalation study. No dose-limiting toxicities (DLTs) were reported during cycle 1. However, following cycle 2 dosing, three patients experienced dose-limiting treatment-emergent adverse events (TEAEs): grade 3 pyrexia and grade 5 hepatic failure (0.19 mg/kg), grade 4 hepatic failure and platelet count decreased (0.25 mg/kg), grade 3 nausea, and grade 4 platelet and neutrophil count decreased (0.25 mg/kg). The RP2D was determined to be 0.064 mg/kg. One patient (0.008 mg/kg) with high baseline GCC expression had an unconfirmed partial response. TAK-164 exhibited a manageable safety profile at 0.064 mg/kg with hepatic toxicity identified as a potential risk. However, the RP2D of 0.064 mg/kg was deemed insufficient to provide clinical benefit, leading to the discontinuation of the program.

### 4.4. Anthracycline

Anthracycline-based drugs, such as doxorubicin [48], exert their therapeutic effect by inducing DNA damage at a structural level, at various stages during DNA replication and transcription. The primary mechanism of action of doxorubicin is topoisomerase II poisoning, where it traps topoisomerase II at the DNA breakage sites by stabilizing the cleavage complexes thereby preventing DNA resealing. Disruption of DNA replication and transcription results in apoptosis-mediated cell death. Additionally, the oxidation of the doxorubicin quinone structure leads to the formation of semi-quinone radicals and, subsequently, superoxide and H_2_O_2_, which elevate oxidative stress and cause further DNA damage [48].

Anthracyclines remain one of the most widely utilized classes of chemotherapeutic agents for treating various malignancies including aggressive non-Hodgkin lymphoma (NHL), acute myeloid leukemia, and breast cancer [49]. However, their use is often limited by cardiotoxicity [50] and the development of resistance.

Few examples of ADCs containing doxorubicin are reported as its high nanomolar antiproliferative potency and strong sensitivity to multidrug resistance (MDR) pumps limited its use. Nonetheless, two doxorubicin ADCs have reached clinical trials: IMMU110 and SGN-15.

IMMU110 (Milatuxumab Doxorubicin) [51], developed by Immunomedics, is a CD74-targeted ADC conjugated to doxorubicin via a hydrazone linker. With an average DAR of 8, achieved upon total disulfide reduction, IMMU110 underwent phase I/II clinical studies administering the drug on days 1, 4, 8, and 11 of a 21-day treatment cycle for multiple myeloma, NHL, CLL, and ALCL. However, the studies have been terminated due to lack of efficacy leading to the abandonment of the ADC.

SGN-15 [52] targets the Lewis Y antigen and contains doxorubicin conjugated to cysteine via an acid labile hydrazone linker. It reached phase II with multiple studies in NSCLC, breast, and prostate cancer. In a randomized Phase II study, 62 patients with recurrent or metastatic NSCLC expressing Lewis Y antigen were treated. Patients were randomized 2:1 to receive SGN-15 200 mg/m^2^/week with docetaxel 35 mg/m^2^/week (Arm A) or docetaxel 35 mg/m^2^/week alone (Arm B) for 6 of 8 weeks. Medial survival time for Arms A and B were 31.4 and 25.3 weeks, with 12-month survivals of 29% and 24%, and 18-month survivals of 18% and 8%, respectively. Despite only mild toxicity in both arms, the company eventually abandoned SGN-15 in favor of other pipeline products.

More recently, PNU-159682 [53] has demonstrated an impressive preclinical profile, with antiproliferative IC50 values in tumor cells ranging from femtomolar to low picomolar and in vivo tumor regression at doses of 15 µg/kg in xenograft mouse models. PNU-159682 is an in vivo metabolite of nemorubicin, an anthracycline discovered by Nerviano Medical Sciences with reduced cardiotoxicity and activity in chemoresistant settings. Although the compound never advanced to clinical trials as a small molecule, it was considered an intriguing payload for ADCs, leading to the development of seven PNU-159682-based ADCs, two of which, NBE-002 [54] and SOT102 reached clinical trials. Both clinically evaluated PNU-159682-derivative ADCs contain an uncleavable linker with a pentaglycine moiety suitable for enzymatic sortase [55] assisted conjugation (SMAC technology) and were as a result highly stable in plasma.

NBE-002 consists of a humanized monoclonal antibody targeting the receptor tyrosine kinase ROR1

In preclinical studies, NBE-002 was evaluated in ROR1-low/-intermediate/-high patient-derived xenograft (PDX) models. NBE-002 was found to display a pronounced effect in TNBC and to induce immunogenic memory, as revealed after tumor re-challenging studies. Despite initiating a phase I/II clinical trial in patients with advanced solid tumors, the trial was recently terminated for undisclosed reasons.

SOT-102, the second PNU-159682-containing ADC, targets Claudin 18.2. It began phase I clinical trials in 2023 to evaluate its safety and efficacy as a monotherapy or in combination with standard-of-care treatments in patients with stomach and pancreas cancer. However, the trial is currently suspended as per the sponsor’s decision.

### 4.5. Duocarmycins

The natural products CC-1065 and duocarmycin SA are irreversible DNA alkylators that exert their effects by docking in the minor groove of DNA. Despite the high in vitro potency of CC-1065, moderate antitumoral in vivo activity and irreversible hepatotoxicity were detected in animal models [56].

Carzelesine ref. [57], one of the synthetic duocarmycin analogs, entered clinical trials following promising preclinical results. However, phase II clinical studies as second- or third-line chemotherapy in patients with breast, ovarian, head and neck cancer, and non-Hodgkin’s lymphoma revealed poor efficacy and a very narrow therapeutic window due to hematotoxicity.

The profile of duocarmycins [58] characterized by picomolar cytotoxic potency across various contexts including chemoresistant, poorly proliferating and tumor stem cells, has garnered significant interest as potential ADC payloads.

Presently, over 10 duocarmycin-based ADCs have been studied with some reaching advanced clinical stage. Notably, SYD-985 (trastuzumab duocarmazine), which contains a DUocarmycin-hydroxyBenzamide Azaindole (DUBA) payload is the most advanced.

The DUBA payload is incorporated in all three duocarmycin-based ADCs currently undergoing clinical trial: HER2-targeted SYD-985/trastuzumab duocarmazine [59], B7-H3-targeted MGC018/Votramimab duocarmazine [60], and cMet-targeted BYON 3521 [61].

Trastuzumab duocarmazine has demonstrated efficacy against patient-derived xenografts (PDXs) with resistance to trastuzumab emtansine (T-DM1). These encouraging results likely stem from the presence of a cleavage point in the linker of trastuzumab duocarmazine targeted for lysosomal release, low levels of HER2 being sufficient for cytotoxicity, and the insensitivity to drug efflux pumps. Trastuzumab duocarmazine has been found to be 3–50 times more effective in cell lines expressing low levels of HER2 compared with T-DM1, whereas the results were similar when HER2 was expressed at high levels. This ADC has been evaluated in three phase I clinical trials including one combination trial with Niraparib in HER2-expressing solid tumors, one phase I/II trial to evaluate the safety and efficacy of sodium thiosulfate eye drops to reduce ocular toxicity, two phase 2 studies in endometrial and breast cancers, and the pivotal phase III TULIP trial. In the TULIP trial, 437 patients with HER-2 positive locally advanced or metastatic breast cancer were treated with either trastuzumab duocarmazine (*n* = 291) at a dose of 1.2 mg/kg every three weeks or physician’s choice chemotherapy (*n* = 146) [62]. Interim results reported [63] that the most common adverse events for trastuzumab duocarmazine were conjunctivitis (38.2%), keratitis (38.2%), and fatigue (33.3%). Dose discontinuation occurred in 35.4% of patients treated with trastuzumab duocarmazine, mainly due to ocular toxicities (20.8%) and respiratory disorders (6.3%).

The study met its primary endpoint of progression-free survival (PFS), demonstrating a statistically significant improvement over physician’s choice along with preliminary supportive overall survival (OS) results. Based on these findings, the FDA accepted a Biological License Application (BLA) for trastuzumab duocarmazine in July 2022. However, in July 2023, Byondis B.V. announced that the FDA had issued a complete response letter (CRL) for BLA suspending the decision on the approval of trastuzumab duocarmazine.

Votramimab duocarmazine (MGC018), an ADC utilizing the DUBA drug linker identical to that in trastuzumab duocarmazine, targets B7-H3, an immune checkpoint antigen highly expressed in most solid tumors. Votramimab duocarmazine has recently entered Phase II/III clinical trials versus androgen-receptor-axis-targeted therapy in patients with metastatic castration-resistant prostate cancer and it is also undergoing clinical phase I/II trials in combination with MGA012, a PD-1-targeting immune checkpoint inhibitor. Preliminary safety reports from these trials indicate that adverse effects are mostly tolerable and the ADC demonstrated early signs of efficacy in some patients.

The generation of ADCs with DUBA presents challenges, as a low DAR of around 2 must be maintained either through site-specific conjugation, as demonstrated by BYON3521 achieving a DAR of 1.8, or via a preparative chromatographic step to eliminate the remaining large amount of unconjugated antibody and the highly conjugated species, thereby avoiding aggregation [64]. The preparative column purification and the low DAR limit DUBA ADCs’ broad applicability, particularly with more hydrophobic antibodies prone to aggregation. Furthermore, some side effects, notably ocular toxicity, observed with DUBA ADCs, could be attributed to the high hydrophobicity of the payload [65].

## 5. Comparative Evaluation of DNA Interacting Payload Features

A recent paper by Lopez de Sa et al. [66] suggested the use of Lipinski’s ‘rule of 5’ to select the ADC payloads most likely to achieve clinical success. Lipinski’s ‘rule of five’ is a qualitative attempt to guide the design of ‘orally deliverable’ compounds and is based on property limits (clogP, molecular weight, and number of hydrogen-bond donors and acceptors) beyond which oral activity is predicted to be poor [67]. In the case of ADCs, the application of this rule will allow better payload druggability predictions including conjugability, through a balanced hydrophobicity–hydrophilicity profile. Furthermore, it can predict the possibility of reaching a higher DAR due to chemical structure flexibility and smaller molecular weight, resulting in an easier allocation of the payload in the antibody structure. Improved druglike properties may also enhance payload permeability through cell membranes to reach its target. Our analysis also incorporates predicted solubility, molecular complexity, and some structural parameters, e.g., sp3 fraction.

A comparative evaluation of tubulin binders, topoisomerase I inhibitors, and DNA-interacting payloads has been performed and results are presented in Table 3, while corresponding payload chemical structures are reported in Supplementary Table S1. The analysis revealed that, consistent with [66] SN38, the sacituzumab govitecan payload, and the deruxtecan, trastuzumab deruxtecan payload are the only payloads fully compliant with the ‘rule of 5’. Conversely, calicheamicin, the payload in the approved ADCs gemtuzumab ozogamicin and inotuzumab ozogamicin, demonstrated the least compliance, satisfying only one out of four Lipinsky’s rules. Calicheamicin exhibited a molecular weight significantly above 500, with 27 hydrogen-bond acceptors and 10 donors far exceeding the limit of 10 and 5, respectively, along with a much larger polar surface area compared with the other evaluated compounds. Additionally, the number of rotatable bonds exceeded the recommended threshold, reaching 22. From this analysis, calicheamicin can be considered poorly compliant for ADC production and this is indeed confirmed by the produced ADCs, which are highly heterogeneous and contain substantial amounts of unconjugated antibody. The suboptimal ADC quality is reflected in the poor pharmacokinetic properties of the ADCs, resulting in a narrow therapeutic window. Furthermore, Calicheamicin’s complexity value of 2500 vastly exceeded that of other payloads under examination, indicating a difficult synthesis and limited possibility of chemical modifications leading to an improved payload.

All the other payloads, while not fully compliant with Lipinsky’s rule, clustered nearby with limited exceptions. Tubulin binders exhibited higher molecular weights, reaching 780 Da with DM4, while PBDs showed the highest number of hydrogen-bond acceptors and seco DUBA was the most hydrophobic compound.

We also assessed the multiparameter optimization (MPO) score [68], which defines druggability in a multidimensional manner, by considering six common physicochemical properties: molecular weight (MW), logP, logD at pH 7.4, topological polar surface area (TPSA), the number of hydrogen-bond donors (HBD), and the pKa of the most basic center.

In the context of ADCs, a higher MPO score could indicate superior payload performance. Indeed, SN38 and deruxtecan, with scores of 4.89 and 3.22, respectively, exhibited the highest MPO in our series, reaffirming their optimal properties for ADC design. PBD and duocarmycin also showed acceptable MPO scores of 3.00 and 2.37, respectively.

Interestingly, the duocarmycin class with the seco-DUBA molecule displayed a profile similar to deruxtecan in most parameters, with high Log P and low solubility being the most critical and distant points.

This is reflected in the quality of produced ADCs, where a tendency for aggregation was observed with increasing DAR, as anticipated. High hydrophobicity could possibly be related to ocular toxicity, the most common side effect for the seco-DUBA-based SYD-985, as hydrophobic molecules can increase macropinocytosis in corneal epithelial cells [69].

Recently, a new thienoduocarmycin payload linker NMS-P945 [70] has been described. The design of NMS-P528, the payload in NMS-P945, includes a pyrrolidine solubilizing moiety to enhance the physicochemical profile and an indolic minor groove binder moiety coupled to a thienoindole scaffold [71] to reach an optimal balance between reactivity and stability, as reported by the Boger group [72]. NMS-P945 was meticulously designed to reduce hydrophobicity, facilitating conjugation with a higher DAR to a wide array of antibodies.

Our analysis was further refined taking into consideration the sensitivity toward drug transporters of the payloads under examination and their antiproliferative activity in tumor cells (Table 4).

As previously noted, an increased expression of drug transporters such as P-gp constitutes the most common cause of chemoresistance to ADC payloads by reducing the intracellular concentration of the free payload.

In the case of ADC payloads, drug transporters not only facilitate the extrusion of the drug from the plasma membrane to the extracellular space but also play a role in drug sequestration within lysosomes, thereby diminishing payload efficacy by hindering its interaction with the target.

Chemoresistance mediated by efflux pumps is prevalent among tubulin-binder payloads, topoisomerase I inhibitors, and most classes of DNA-interacting payloads. Duocarmycins and PNU-159682 are indeed the only DNA-interactive molecules reported not to be sensitive to this mechanism. This distinction highlights these two classes as the most promising candidates for overcoming resistance issues in the ADC field.

In a recent paper, Colombo et al. [73] demonstrated that the tolerated doses of different ADCs with identical payloads are comparable and not significantly different from those of related small molecules. Nevertheless, when administered at or near the maximum tolerated dose, certain ADCs exhibit enhanced efficacy compared to their corresponding small molecule. Further elaborating on this concept, we compared the antiproliferative activity in tumor cells of the different payloads (Table 4) and we concluded that payloads with potency ranging from double-digit picomolar to single-digit nanomolar could be considered the most appropriate for successful ADC development. This is indeed the payload potency range of 11 out of the 12 currently FDA-approved ADCs applying current conjugation technologies. The three explored chemical classes showing higher potency and reaching femtomolar activity, PDB, IGN, and PNU-159682, are associated with higher numbers of serious adverse events strongly limiting their clinical development. In Table 4, MTD in patients for cytotoxic small molecules and their corresponding ADCs is presented with a focus on DNA-interacting molecules compared to tubulin binders and topoisomerase I inhibitors. This analysis, in line with Colombo et al. considerations, reveals a certain correlation between small molecule MTD and corresponding ADCs, suggesting a payload-related effect on MTD. This finding further supports the preference for medium-potency cytotoxic drugs for ADC development.

## 6. Conclusions and Future Perspectives

ADCs represent a significant class of cancer therapeutics, with several FDA-approved ADCs available for the treatment of various cancers and many more molecules undergoing clinical evaluation. Inherent and acquired drug resistance, however, remain major challenges to successful treatment.

The recent success of topoisomerase I inhibitors in the approved ADCs reinforces the idea that new payloads with different mechanisms of action can be effective in tumor settings where microtubule-binding agents failed when bound to the same targeting mAb. Consequently, ADCs targeting antibodies previously deemed unsuccessful in clinical trials can be reconsidered in light of new payloads and linkers, potentially opening new tumor indications, and overcoming the limitations of first-generation ADCs against the same targets.

Our comparative analysis aimed to deeply explore if there is still a potential for DNA-interacting agents as ADC payloads. Despite their early exploration as payloads due to favorable properties in terms of antiproliferative activity throughout the cell cycle, including poorly proliferating or tumor stem cells and, in some cases, activity in chemoresistant settings, many companies have struggled to develop viable payload linkers from these potent cytotoxic DNA-interacting molecules.

Our analysis showed that the future of ADC payloads lies in those with favorable physicochemical and conformational properties well described by MPO scoring and antiproliferative potency in the high picomolar to low nanomolar range.

Adhering to these parameters typically facilitates easy conjugation to a wide range of antibodies, expedites preclinical development, and ensures manageable toxicity if any payload molecule is released from the antibody.

Many studied DNA-interacting agents do not comply with these parameters. Their unfavorable physicochemical properties, challenging synthetic processes, and extreme cytotoxic potency must be managed to produce viable ADCs. The recent approval of the PBD-based loncastuximab tesirine, along with the successful late-stage clinical trials of other molecules, indicates that successful ADCs can indeed be generated with DNA-interacting payloads by carefully modulating their physicochemical properties and selecting the proper targeting agents. Clinical studies on PBD-dimer- and calicheamicin-containing ADCs showed that a clinical development plan with dose scheduling optimization and careful patient selection is crucial to minimize delayed toxicity. It is worth noting that in PBD-dimer ADC clinical studies, responses have been observed in some patients at very low starting doses, possibly indicating a peculiar sensitivity of some patients to the treatment. This finding suggests that the introduction of predictive biomarkers suitable for patient stratification could also help in the successful development of next-generation ADCs.

Among different DNA-interacting chemical classes, our evaluation indicated anthracyclines and duocarmycins as the classes with more potential for future development.

Anthracyclines, along with deruxtecan (a camptothecin derivative), are the only payloads also approved as small molecule drugs for anticancer therapy, implying they possess suitable physicochemical properties, permeability, and metabolic profiles for patient administration. While approved anthracycline small molecules exhibit antiproliferative potency that is not ideally suited for ADC development, the discovery of PNU-159682 suggests a potential for potency enhancement. Studies of molecular design aimed at potency optimization and physicochemical properties enhancement are ongoing, as exemplified by recent works by Orsini et al. [74] and other groups, opening up the possibility of next-generation anthracycline-based ADCs.

Duocarmycins also represent an expandable class for future ADCs. These molecules are highly attractive due to their insensitivity to MDR pumps and appropriate cytotoxic potency range.

Despite historical biases against their use due to poor physicochemical profiles and challenging synthetic processes, the recent introduction of the NMS-P945 thienoindole payload [70] demonstrates that modifying the duocarmycin chemical scaffold through structure-based drug design can balance antiproliferative potency, molecular reactivity, and physicochemical properties.

Indeed, in silico analysis of NMS-P528, the payload included in NMS-P945, indicated an increased solubility of one log compared to seco DUBA, reaching 3.5 × 10^−5^ M, a polar surface area comparable to SN38 and an improved MPO of 2.57, indicating better druggability than competitor duocarmycin payloads. This once more reflected the better conjugability of this molecule, which easily reached a DAR of around 4 with a large number of different monoclonal antibodies.

Overall, this review provides a comprehensive and critical analysis of the current state of ADCs with DNA-damage mechanisms, offering valuable insights and potential strategies for optimizing current molecules.

The availability of payloads with improved properties conjugated to appropriate targeting agents in a patient population selected by predictive biomarker analysis will potentially open new tumor indications, overcoming the limitations of first-generation ADCs.

## Figures and Tables

**Table 1:** FDA regulatory approved ADCs.

Drug Names	Year of Approval	Target	Linker	Linker Catabolism	Payload	DAR	Conjugation Amino Acid	Mechanism of Action	Approved Disease Indications
Mylotarg™; Gemtuzumab ozogamicin	2000	CD33	AcBut acyl hydrazone-disulfide	Cleavable	Calicheamicin	2–3	Lysine	DNA binder	Acute Myeloid Leukemia
Adcetris™; Brentuximab vedotin	2011	CD30	Valine-Citrulline	Cleavable	MMAE	4	Cysteine	Tubulin binder	Classical Hodgkin Lymphoma; Cutaneous T-Cell Lymphoma; PTCL; sALCL
Kadcyla™; T-DM1; Trastuzumab emtansine	2013	HER-2	SMCC	Non-Cleavable	DM1	3.5	Lysine	Tubulin binder	HER2 Positive Early Breast Cancer; HER2+ Metastatic Breast Cancer
Besponsa™; Inotuzumab ozogamicin	2017	CD22	AcBut acyl hydrazone-disulfide	Cleavable	Calicheamicin	2–3	Lysine	DNA binder	B-cell Precursor (Ph+ Negative) Acute Lymphoblastic Leukemia
PADCEV™; Enfortumab vedotin	2019	Nectin-4	Valine-Citrulline	Cleavable	MMAE	4	Cysteine	Tubulin binder	Advanced Urothelial Cancer; Metastatic Urothelial Cancer
POLIVY™; Polatuzumab vedotin	2019	CD79b	Valine-Citrulline	Cleavable	MMAE	3.5	Cysteine	Tubulin binder	Diffuse Large B-cell lymphoma
ENHERTU™; Trastuzumab deruxtecan	2019	HER-2	GGFG	Cleavable	DXd	8	Cysteine	TOPO I inhibitor	HER2 Mutant metastatic NSCLC HER-2 Positive Advanced/Metastatic Gastric Cancer; HER2-Positive Advanced/metastatic Gastroesophageal Cancer; Metastatic HER2 Positive Breast Cancer;
Trodelvy™; Sacituzumab govitecan	2020	TROP-2	CL2A	Cleavable	SN-38	7.6	Cysteine	TOPO I inhibitor	Advanced/metastatic TNBC Advanced/Metastatic Urothelial Cancer;
BLENREP™; Belantamab mafodotin	2020	BCMA	maleimido-caproyl	Non-Cleavable	MMAF	4	Cysteine	Tubulin binder	Myeloma/Multiple Myeloma/Kahler’s disease/Myelomatosis
ZYNLONTA™ Loncastuximab Tesirine	2021	CD19	Valine-Alanine	Cleavable	SG3199 (PBD)	2.3 ± 0.3	Cysteine	DNA binder	Diffuse Large B-Cell Lymphoma
TIVDAK™ Tisotumab vedotin	2021	TF	Valine-Citrulline	Cleavable	MMAE	4	Cysteine	Tubulin binder	Metastatic Cervical Cancer; Recurrent Cervical Cancer
ELAHERE™ Mirvetuximab soravtansine	2022	FOLRA	Sulfo-SPDB	Cleavable	DM4	3.4	Lysine	Tubulin binder	Platinum-Resistant Ovarian Cancer; Platinum-Resistant Fallopian Tube Carcinoma; Primary Peritoneal Cancer

**Table 2:** Properties of most-advanced ADCs containing DNA-binder payloads versus Tubulin binders and topoisomerase I inhibitors.

Most Advanced ADC	Most Advanced Stage	Payload Chemical Class	Payload Mechanism of Action	Linker Catabolism	DAR	Conjugation Amino Acid	Target	Most Common Adverse Events
brentuximab vedotin	FDA approved	Auristatin	Tubulin binder	cleavable	4	Cysteine	CD30	Peripheral neuropathy
enfortumab vedotin	FDA approved	Auristatin	Tubulin binder	cleavable	4	Cysteine	Nectin 4	Peripheral neuropathy, Skin reactions
disitamab vedotin	China approved	Auristatin	Tubulin binder	cleavable	4	Cysteine	HER2	ALT, AST elevation
tisitamab vedotin	FDA approved	Auristatin	Tubulin binder	cleavable	4	Cysteine	TF	Peripheral neuropathy, bleeding
polatuzumab vedotin	FDA approved	Auristatin	Tubulin binder	cleavable	3.8	Cysteine	CD79B	Peripheral neuropathy Anemia, neutropenia
Trastuzumab emtansine	FDA approved	Maytansine	Tubulin binder	uncleavable	3.5	Lysine	HER2	Gastrointestinal disorders, thrombocytopenia
Mirvetuximab sorvantansine	FDA approved	Maytansine	Tubulin binder	uncleavable	3.5	Lysine	FOLRA	Ocular Tox, pneumonia Peripheral neuropathy
Trastuzumab deruxtecan	FDA approved	Camptothecin	Topoisomerase I inhibitor	cleavable	8	Cysteine	HER2	Gastrointestinal disorders, ILD
Sacituzumab govitecan	FDA approved	Camptothecin	Topoisomerase I inhibitor	cleavable	8	Cysteine	TROP2	Neutropenia, gastrointestinal disorders
Gemtuzumab ozogamicin	FDA approved	Calicheamicin	Minor groove binder and DNA double-strand break inducer	cleavable	2–3	Lysine	CD33	Neutropenia VOD, hepatic failure
Inotuzumab ozogamicin	FDA approved	Calicheamicin	Minor groove binder and DNA double-strand break inducer	cleavable	2–3	Lysine	CD22	Neutropenia VOD hepatic failure
Loncastuximab Tesirine	FDA approved	PBD dimer	Minor groove DNA binder and Guanine crosslinker	cleavable	2.3	Cysteine	CD19	Respiratory disorders
Rovalpituzumab tesirine	Phase III	PBD dimer	DNA crosslinker	cleavable	2	Cysteine	DLL3	Respiratory disorders, neutropenia, thrombocytopenia
Pivekimab Sunirine	Phase II	IGN	DNA alkylating	cleavable		Cysteine	CD123	Respiratory disorders, neutropenia, thrombocytopenia
TAK-164	Phase II	IGN	DNA alkylating	cleavable		Cysteine	GCC	Thrombocytopenia hepatic tox
NBE-002	Phase I	Anthracycline	Topo II inhibition and DNA intercalation	uncleavable	2	C terminus	ROR1	No data
SOT102	Phase I	Anthracycline	Topo II inhibition and DNA intercalation	uncleavable	2	C terminus	CLDN18.2	No data
SGN-15	Phase II	Anthracycline	Topo II inhibition and DNA intercalation	cleavable		Cysteine	Lewis Y antigen	Gastrointestinal respiratory disorders
IMMU 110	Phase I/II	Anthracycline	Topo II inhibition and DNA intercalation	cleavable	8	Cysteine	CD74	
Trastuzumab duocarmazine	Phase III	Duocarmycin	DNA minor groove binder and alkylating agent	cleavable	2.8	Cysteine	HER2	Ocular tox, respiratory disorders
MGC018	Phase II	Duocarmycin	DNA minor groove binder and alkylating agent	cleavable	2.8	Cysteine	B7-H3	Skin disorders, neutropenia, respiratory disorders

**Table 3:** Physicochemical property comparison among DNA-binder, Tubulin-binder, and topoisomerase-I-inhibitor-based payloads.

Drug Name	MOA	MW	Complexity	ACD_ LogP	H-Bond Donors	H-Bond Acceptors	Rotatable Bonds	PSA	PSA_ SASA	MPO	sp3 Fractions	Sol (mol/L)
Desired Features		<500		<5	<5	<10	<10	<140		>3	>0.42	0.001
MMAE	tubulin binder	718.99	1100	3.82	4	8	20	154.11	0.8	1.81	0.74	1.00 × 10^−2^
DM1	tubulin binder	738.29	1340	3.87	2	11	8	195.27	0.74	2.14	0.6	4.40 × 10^−7^
DM4	tubulin binder	780.37	1430	4.81	2	11	9	195.27	0.74	1.6	0.63	2.80 × 10^−7^
SN38	topo I inhibitor	392.4	882	2.11	2	7	2	99.96	0.64	4.89	0.32	3.50 × 10^−3^
DXd	topo I inhibitor	493.48	1080	1.76	3	8	3	129.06	0.65	3.22	0.38	3.90 × 10^−4^
N acetyl calicheamicin	DNA damage	1478.44	2500	6.13	10	27	26	447.74	0.76	1	0.66	4.70 × 10^−6^
SG3199 (PBD)	DNA crosslinker	602.68	1080	1.8	2	24	10	122.16	0.85	3.5	0.42	7.70 × 10^−5^
Sunirine	DNA alkylator	788.84	na	1.05	5	9	9	193.2	0.72	3	0.24	3.50 × 10^−2^
PNU	topo II inhibitor	642.63	1200	3.64	4	14	6	191.95	0.7	2.11	0.53	3.40 × 10^−6^
Seco DUBA	MGBAA	526.97	885	3.76	3	5	4	107.16	0.63	2.37	0.14	4.50 × 10^−6^

**Table 4:** Cellular potency of payloads and properties of corresponding most advanced ADCs.

Payload Name	MOA	Efflux Pumps Sensitivity	Potency M	MTD Free Toxin (mg/kg)	Most Advanced ADC	Bystander Effect	MTD ADC Toxin Normalized (mg/kg)
MMAE	tubulin binder	++	10^−11^–10^−10^	Dolastatin 0.008	Brentuximab Vedotin	Yes	0.032 Q3W
					Enfortumab Vedotin	Yes	0.022 D1, 8, 15, 28 days cycle
					Disitamab Vedotin	Yes	0.038 Q2W
					Tisitamab Vedotin	Yes	0.037 Q3W
					Polatuzumab Vedotin	Yes	0.034 Q3W
DM1	tubulin binder	++	10^−11^–10^−10^	Maytansine 0.054 Q3W	TDM1 (Kadcyla)	No	0.062Q3W
DM4	tubulin binder	++	10^−11^–10^−10^	Maytansine 0.054 Q3W	Mirvetuximab soravtansine (Elahere)	No	0.11 Q3W
SN38	topo I inhibitor	++	10^−9^–10^−8^	Irinotecan 0.12 QW	Sacituzumab govitecan (Trodelvy)	Yes	0.19 D1, 8, 21 Days cycle
DXd	topo I inhibitor	+	10^−10^–10^−9^	Topotecan 0.20 QDX5 every three weeks	Trastuzumab deruxtecan (Enhertu)	Yes	0.14 Q3W
N acetyl calicheamicin	DNA damage	++	10^−11^–10^−10^	not available	Gemtuzumab ozogamicine (Mylotarg)	Yes	0.00087 fractionated dose on day 1, 8 15
					Inotuzumab ozogamicine (Besponsa)	Yes	
SG3199 (PBD)	DNA crosslinker	+	10^−13^–10^−12^	SJG-136 0.0012 Q3W	Zynlonta	Yes	0.0013 Q3W
					Rovalpizumab tesirine	Yes	
Sunirine	DNA alkylator	+	10^−13^–10^−12^	SJG-136 0.0012 Q3W	Pivekimab sunirine	Yes	0.00042 Q3W
					TAK164	Yes	
PNU	topo II inhibitor, DNA intercalation	-	10^−13^–10^−12^	Nemorubicin 0.022 Q4W	NBE002	Yes	Not reported
					SOT102	Yes	Not reported
Seco-DUBA	MGBAA	-	10^−11^–10^−10^	Carzelesine 0.0081Q4W	SYD985	Yes	0.011 Q3W
					MGC018	Yes	

- No sensitivity. + medium sensitivity. ++ high sensitivity.

## Data Availability

Data sharing is not applicable.

## Supplementary Materials

The following supporting information can be downloaded at: https://www.mdpi.com/article/10.3390/ph17101338/s1, Table S1: Chemical structure of ADC payload linkers
