# Supplementary material for: Present Scenario and Future Landscape of Payloads for ADCs: Focus on DNA-Interacting Agents
Source: Pharmaceuticals (Basel). 2024 Oct 7;17(10):1338. doi: 10.3390/ph17101338 (PMC11510327; doi:10.3390/ph17101338)
Supplement: Supplementary file 1 [file pharmaceuticals-17-01338-s001.zip › pharmaceuticals-3173717-supplementary.pdf]

Table S1

Chemistry	drug name	MOA
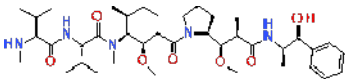	MMAE	tubulin binder
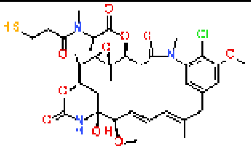	DM1	tubulin binder
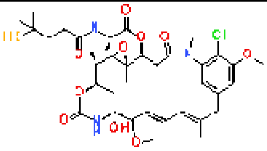	DM4	tubulin binder
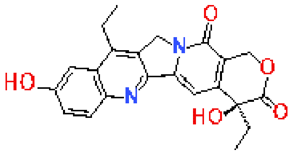	SN38	topo I inhibitor
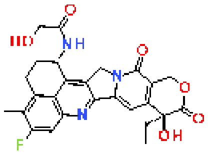	DXd	topo I inhibitor
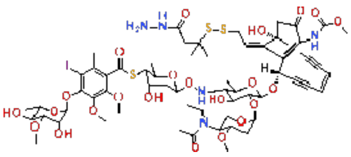	N acetyl calicheamicin	DNA damage
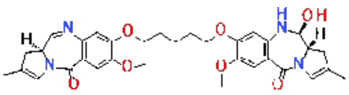	SG3199 (PBD)	DNA crosslinker
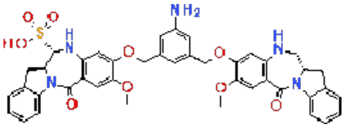	Sunirine	DNA alkylator
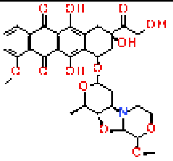	PNU	topo II inhibitor
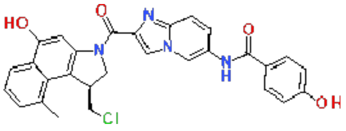	Seco DUBA	MGBAA
